# Supplementary material for: Prognostic roles of diabetes mellitus and hypertension in advanced hepatocellular carcinoma treated with sorafenib
Source: PLoS One. 2020 Dec 31;15(12):e0244293. doi: 10.1371/journal.pone.0244293 (PMC7775090; doi:10.1371/journal.pone.0244293)
Supplement: S1 Table — (PDF) [file pone.0244293.s002.pdf]

S1 Table. Patient characteristics in each separate group of the DM cohort (diabetic patients with or without HTN, i.e. the combination cohort of DM-only and DM+HTN groups; n=196).

	Separate groups: divided by the types of diabetes medications			p-value		
	I: metformin (n=63)	II: non-metformin OHA (n=104)	III: RI/NPH (n=29)	I vs. II	I vs. III	II vs. III
**Baseline characteristics**						
Male†	37 (58.7%)	86 (82.7%)	19 (65.5%)	0.001*	0.647	0.069
Age, mean (range)‡	65.89±8.99 (44-84)	64.03±9.86 (36-84)	65.07±8.70 (49-85)	0.224	0.682	0.608
HTN	37 (58.7%)	54 (51.9%)	14 (48.3%)	0.426	0.375	0.834
HBV or HCV infection†						
HBV only	20 (31.7%)	35 (33.7%)	12 (41.4%)	0.866	0.480	0.512
HCV only	8 (12.7%)	11 (10.6%)	2 (6.9%)	0.802	0.496	0.733
HBV+HCV	2 (3.2%)	1 (1.0%)	0 (0.0%)	0.557	1.000	1.000
None	33 (52.4%)	57 (54.8%)	15 (51.7%)	0.873	1.000	0.835
Liver cirrhosis†	55 (87.3%)	83 (79.8%)	26 (89.7%)	0.292	1.000	0.283
Tumor site†						
Intra-hepatic venous invasion only	23 (36.5%)	40 (38.5%)	13 (44.8%)	0.870	0.495	0.668
Extra-hepatic metastases only	32 (50.8%)	54 (51.9%)	13 (44.8%)	1.000	0.657	0.535
lymph nodes	10 (15.9%)	13 (12.5%)	5 (17.2%)	0.644	1.000	0.543
lung	17 (27.0%)	24 (23.1%)	6 (20.7%)	0.583	0.610	1.000
adrenal gland	2 (3.2%)	2 (1.9%)	0 (0.0%)	0.633	1.000	1.000
bone	6 (9.5%)	6 (5.8%)	2 (6.9%)	0.372	1.000	1.000
other[a]	1 (1.6%)	7 (6.7%)	1 (3.4%)	0.261	0.533	1.000
multi-organ	4 (6.3%)	12 (11.5%)	2 (6.9%)	0.416	1.000	0.734
Intra-hepatic venous invasion plus extra-hepatic metastases	8 (12.7%)	10 (9.6%)	3 (10.3%)	0.609	1.000	1.000
Blood pressure (mmHg)[b]‡						
Systolic, mean (range)	133.03±16.46 (87-170)	132.49±18.39 (93-190)	135.10±19.27 (110-187)	0.848	0.597	0.504
Diastolic, mean (range)	76.68±11.78 (56-114)	75.74±10.25 (44-99)	76.41±9.12 (61-97)	0.587	0.914	0.749
Glucose, mean (mg/dL) (range)[b]‡	167.33±54.15 (97-317)	162.58±66.08 (73-443)	188.55±71.04 (66-354)	0.631	0.118	0.068
HbA1c, mean (%) (range)[b]‡	7.30±1.82 (5.1-13.3)	7.02±1.49 (4.8-12.5)	8.31±2.13 (5.8-14.2)	0.276	0.022*	0.004*

AFP, mean (ng/mL) (range)‡	2652.52±8621.06 (1.55-54001.00)	4026.30±11443.21 (1.49-54001.00)	3278.68±10639.85 (0.91-54001.00)	0.412	0.765	0.753
**Sorafenib duration, median (month) (range)** [c]§	5.00 (0.27-50.17)	3.97 (0.13-77.63)	2.83 (0.17-27.57)	0.356	0.157	0.361
**On-sorafenib HbA1c, mean (%) (range)**[d]‡	7.15±1.42 (5.2-10.6)	7.12±1.44 (4.9-11.2)	7.84±2.06 (4.8-12.2)	0.924	0.110	0.088
**Events during the study**†						
Expired	51 (81.0%)	93 (89.4%)	24 (82.8%)	0.164	1.000	0.341
PD	41 (65.1%)	66 (63.5%)	17 (58.6%)	0.869	0.644	0.668

Abbreviation: DM, diabetes mellitus; HTN, hypertension; OHA, oral hypoglycemic agent; RI, regular insulin; NPH, neutral protamine hagedorn; HBV, hepatitis B virus; HCV, hepatitis C virus; HbA1c, hemoglobin A1c; AFP, alpha-fetoprotein; PD, progression disease.

Note: [a]Tumor involvement of an extra-hepatic organ other than lymph nodes, lung, adrenal gland and bone. [b]To eliminate the bias, data of baseline blood pressure (BP), HbA1c and glucose levels for each patient were determined by calculating the mean values of BP, HbA1c and glucose levels measured multiple (two or three) times at baseline. [c]The duration of sorafenib therapy depended on the therapeutic response evaluated every two to three months since baseline. Patients allowed to continue sorafenib therapy were those with benign response [complete response (CR), partial response (PR) or stable disease (SD) rated by modified Response Evaluation Criteria in Solid Tumors (mRECIST)] and the maintenance of Child-Pugh class A. [d]Data of on-sorafenib HbA1c level derived from calculating the mean value of serum HbA1c level measured multiple times during sorafenib therapy. †Fisher exact test. ‡independent-samples t-test. §Mann-Whitney U test. *A p-value below 0.05 was considered statistically significant.
