# Supplementary material for: Prognostic roles of diabetes mellitus and hypertension in advanced hepatocellular carcinoma treated with sorafenib
Source: PLoS One. 2020 Dec 31;15(12):e0244293. doi: 10.1371/journal.pone.0244293 (PMC7775090; doi:10.1371/journal.pone.0244293)
Supplement: S2 Table — (PDF) [file pone.0244293.s003.pdf]

**S2 Table. Assessing the correlation between hemoglobin A1c (HbA1c) level and sorafenib duration (an indicator of treatment response) in the DM cohort (diabetic patients with or without HTN, i.e. the combination cohort of DM-only and DM+HTN groups; n=196): a Cox regression model.**

| variable | case number | Univariate analysis | | Multivariate analysis[a] | |
|---|---|---|---|---|---|
| | | **HR (95% CI)** | **p-value** | **HR (95% CI)** | **p-value** |
| **Baseline characteristics** | | | | | |
| Diabetes medication | | | | | |
| Metformin | | | | | |
| No | 133 | Ref | | Ref | |
| Yes | 63 | 0.859 (0.617-1.197) | 0.369 | 0.916 (0.540-1.552) | 0.744 |
| Non-metformin OHA | | | | | |
| No | 92 | Ref | | Ref | |
| Yes | 104 | 1.051 (0.773-1.430) | 0.749 | 0.904 (0.550-1.484) | 0.689 |
| RI/NPH | | | | | |
| No | 167 | Ref | | | |
| Yes | 29 | 1.196 (0.775-1.846) | 0.420 | | |
| HTN | | | | | |
| No | 91 | Ref | | Ref | |
| Yes | 105 | 0.910 (0.671-1.233) | 0.542 | 0.906 (0.658-1.248) | 0.547 |
| Sex | | | | | |
| Female | 54 | Ref | | Ref | |
| Male | 142 | 1.115 (0.788-1.577) | 0.538 | 1.175 (0.793-1.741) | 0.422 |
| Age | | | | | |
| <65 | 95 | Ref | | Ref | |
| ≥65 | 101 | 1.172 (0.865-1.589) | 0.306 | 1.167 (0.831-1.639) | 0.373 |
| HBV and/or HCV infection | | | | | |
| No | 105 | Ref | | Ref | |
| Yes | 91 | 0.664 (0.488-0.903) | 0.009* | 0.718 (0.518-0.995) | 0.047* |
| Liver cirrhosis | | | | | |
| No | 32 | Ref | | Ref | |
| Yes | 164 | 0.799 (0.529-1.205) | 0.284 | 0.859 (0.559-1.322) | 0.490 |
| Intra-hepatic venous invasion | | | | | |
| No | 99 | Ref | | Ref | |
| Yes | 97 | 1.040 (0.768-1.408) | 0.800 | 1.125 (0.793-1.596) | 0.510 |

| | | | | | |
|---|---|---|---|---|---|
| Multi-organ metastases | | | | | |
| No | 178 | Ref | | Ref | |
| Yes | 18 | 1.635 (0.985-2.712) | 0.057 | 1.862 (1.060-3.273) | 0.031* |
| Intra-hepatic venous invasion plus extra-hepatic metastases | | | | | |
| No | 175 | Ref | | Ref | |
| Yes | 21 | 0.935 (0.572-1.530) | 0.790 | 0.821 (0.469-1.439) | 0.491 |
| AFP | | | | | |
| ≥400 ng/mL | 61 | Ref | | Ref | |
| <400 ng/mL | 135 | 0.720 (0.522-0.992) | 0.044* | 0.717 (0.505-1.017) | 0.062 |
| HbA1c[b] | | | | | |
| ≥7 % | 86 | Ref | | Ref | |
| <7 % | 110 | 0.953 (0.702-1.292) | 0.755 | 0.979 (0.680-1.410) | 0.908 |
| **On-sorafenib HbA1c**[c] | | | | | |
| ≥7 % | 93 | Ref | | Ref | |
| <7 % | 103 | 0.865 (0.638-1.172) | 0.348 | 0.810 (0.564-1.163) | 0.254 |

Abbreviation: HR, hazard ratio; CI, confidence interval; Ref, reference variable; OHA, oral hypoglycemic agent; RI, regular insulin; NPH, neutral protamine hagedorn; HTN, hypertension; HBV, hepatitis B virus; HCV, hepatitis C virus; AFP, alpha-fetoprotein; HbA1c, hemoglobin A1c.

**Note:** [a]To confirm the correlation between each variable and sorafenib duration, all variables except the use of RI/NPH (yes/no) were entered into multivariate analysis. The use of RI/NPH (yes/no) was a covariate constant or linearly dependent to variables of metformin use (yes/no) and non-metformin OHA prescription (yes/no). Therefore, this variable was not entered into multivariate analysis when the other two were entered. [b]To eliminate the bias, data of baseline HbA1c level for each patient were determined by calculating the mean value of HbA1c level measured multiple (two or three) times at baseline. [c]Data of on-sorafenib HbA1c level derived from calculating the mean value of serum HbA1c level measured multiple times during sorafenib therapy. *A p-value below 0.05 was considered statistically significant.
