# Supplementary material for: Prognostic roles of diabetes mellitus and hypertension in advanced hepatocellular carcinoma treated with sorafenib
Source: PLoS One. 2020 Dec 31;15(12):e0244293. doi: 10.1371/journal.pone.0244293 (PMC7775090; doi:10.1371/journal.pone.0244293)
Supplement: S3 Table — (PDF) [file pone.0244293.s004.pdf]

S3 Table. Survival differences among separate groups of the DM cohort (diabetic patients with or without HTN, i.e. the combination cohort of DM-only and DM+HTN groups; n=196).

	**OS, median (95% CI) (month)†**	**PFS, median (95% CI) (month)†**
**Metformin (n=63)**	12.60±2.17 (8.34-16.86)	8.17±1.53 (5.16-11.17)
**Non-metformin OHA (n=104)**	10.80±1.20 (8.44-13.16)	5.67±1.57 (2.59-8.75)
**RI/NPH (n=29)**	15.20±4.45 (6.49-23.91)	7.17±2.04 (3.17-11.16)
	**Log-rank test**	
	**OS**	**PFS**
**Metformin vs. Non-metformin OHA**	p=0.831	p=0.704
**Metformin vs. RI/NPH**	p=0.494	p=0.607
**Non-metformin OHA vs. RI/NPH**	p=0.465	p=0.400

Abbreviation: OS, overall survival; CI, confidence interval; PFS, progression-free survival; OHA, oral hypoglycemic agent; RI, regular insulin; NPH, neutral protamine hagedorn. †Kaplan-Meier method: OS and PFS were shown as median ± standard error with 95% CI. *A p-value below 0.05 was considered statistically significant.
