# Supplementary material for: Prognostic roles of diabetes mellitus and hypertension in advanced hepatocellular carcinoma treated with sorafenib
Source: PLoS One. 2020 Dec 31;15(12):e0244293. doi: 10.1371/journal.pone.0244293 (PMC7775090; doi:10.1371/journal.pone.0244293)
Supplement: S4 Table — (PDF) [file pone.0244293.s005.pdf]

S4 Table. Cox regression of overall survival (OS) in the DM cohort (diabetic patients with or without HTN, i.e. the combination cohort of DM-only and DM+HTN groups; n=196).

variable	case number	**Univariate analysis**		**Multivariate analysis**[a]	
		**HR (95% CI)**	**p-value**	**HR (95% CI)**	**p-value**
**Baseline characteristics**					
Diabetes medication					
Metformin					
No	133	Ref		Ref	
Yes	63	1.004 (0.720-1.399)	0.983	1.284 (0.759-2.174)	0.351
Non-metformin OHA					
No	92	Ref		Ref	
Yes	104	1.083 (0.798-1.471)	0.609	1.182 (0.723-1.933)	0.504
RI/NPH					
No	167	Ref			
Yes	29	0.854 (0.554-1.317)	0.476		
HTN					
No	91	Ref		Ref	
Yes	105	1.063 (0.785-1.441)	0.692	1.035 (0.747-1.435)	0.836
Sex					
Female	54	Ref		Ref	
Male	142	0.961 (0.679-1.360)	0.823	1.005 (0.676-1.493)	0.981
Age					
<65	95	Ref		Ref	
≥65	101	1.216 (0.897-1.646)	0.207	1.136 (0.806-1.600)	0.467
HBV and/or HCV infection					
No	105	Ref		Ref	
Yes	91	0.651 (0.478-0.886)	0.006*	0.725 (0.521-1.008)	0.056
Liver cirrhosis					
No	32	Ref		Ref	
Yes	164	0.820 (0.544-1.236)	0.343	0.971 (0.626-1.505)	0.895
Intra-hepatic venous invasion					
No	99	Ref		Ref	
Yes	97	1.188 (0.877-1.609)	0.265	1.339 (0.945-1.897)	0.101
Multi-organ metastases					

No	178	Ref		Ref	
Yes	18	1.830 (1.103-3.036)	0.019*	2.253 (1.272-3.990)	0.005*
Intra-hepatic venous invasion plus extra-hepatic metastases					
No	175	Ref		Ref	
Yes	21	1.125 (0.689-1.838)	0.638	0.820 (0.464-1.450)	0.495
AFP					
≥400 ng/mL	61	Ref		Ref	
<400 ng/mL	135	0.669 (0.486-0.923)	0.014*	0.639 (0.448-0.910)	0.013*
HbA1c[b]					
≥7 %	86	Ref		Ref	
<7 %	110	1.080 (0.796-1.465)	0.622	1.080 (0.747-1.562)	0.683
**On-sorafenib HbA1c**[c]					
≥7 %	93	Ref		Ref	
<7 %	103	0.871 (0.642-1.180)	0.372	0.726 (0.499-1.055)	0.093

Abbreviation: HR, hazard ratio; CI, confidence interval; Ref, reference variable; OHA, oral hypoglycemic agent; RI, regular insulin; NPH, neutral protamine hagedorn; HTN, hypertension; HBV, hepatitis B virus; HCV, hepatitis C virus; AFP, alpha-fetoprotein; HbA1c, hemoglobin A1c.

Note: [a]To confirm the correlation between each variable and OS, all variables except the use of RI/NPH (yes/no) were entered into multivariate analysis. The use of RI/NPH (yes/no) was a covariate constant or linearly dependent to variables of metformin use (yes/no) and non-metformin OHA prescription (yes/no). Therefore, this variable was not entered into multivariate analysis when the other two were entered. [b]To eliminate the bias, data of baseline HbA1c level for each patient were determined by calculating the mean value of HbA1c level measured multiple (two or three) times at baseline. [c]Data of on-sorafenib HbA1c level derived from calculating the mean value of serum HbA1c level measured multiple times during sorafenib therapy. *A p-value below 0.05 was considered statistically significant.
