# Supplementary material for: Prognostic roles of diabetes mellitus and hypertension in advanced hepatocellular carcinoma treated with sorafenib
Source: PLoS One. 2020 Dec 31;15(12):e0244293. doi: 10.1371/journal.pone.0244293 (PMC7775090; doi:10.1371/journal.pone.0244293)
Supplement: S5 Table — (PDF) [file pone.0244293.s006.pdf]

S5 Table. Cox regression of progression-free survival (PFS) in the DM cohort (diabetic patients with or without HTN, i.e. the combination cohort of DM-only and DM+HTN groups; n=196).

variable	case number	Univariate analysis		Multivariate analysis[a]	
		HR (95% CI)	p-value	HR (95% CI)	p-value
**Baseline characteristics**					
Diabetes medication					
Metformin					
No	133	Ref		Ref	
Yes	63	0.976 (0.700-1.361)	0.886	1.278 (0.754-2.164)	0.362
Non-metformin OHA					
No	92	Ref		Ref	
Yes	104	1.114 (0.821-1.511)	0.489	1.192 (0.732-1.942)	0.481
RI/NPH					
No	167	Ref			
Yes	29	0.846 (0.548-1.306)	0.451		
HTN					
No	91	Ref		Ref	
Yes	105	0.985 (0.727-1.335)	0.923	0.924 (0.671-1.274)	0.631
Sex					
Female	54	Ref		Ref	
Male	142	1.060 (0.750-1.500)	0.740	1.089 (0.733-1.616)	0.674
Age					
<65	95	Ref		Ref	
≥65	101	1.164 (0.859-1.577)	0.327	1.070 (0.766-1.493)	0.692
HBV and/or HCV infection					
No	105	Ref		Ref	
Yes	91	0.603 (0.443-0.821)	0.001*	0.636 (0.459-0.879)	0.006*
Liver cirrhosis					
No	32	Ref		Ref	
Yes	164	0.763 (0.505-1.152)	0.198	0.827 (0.535-1.278)	0.392
Intra-hepatic venous invasion					
No	99	Ref		Ref	
Yes	97	1.082 (0.799-1.464)	0.612	1.151 (0.810-1.636)	0.433
Multi-organ metastases					

No	178	Ref		Ref	
Yes	18	1.867 (1.125-3.100)	0.016*	2.162 (1.235-3.787)	0.007*
Intra-hepatic venous invasion plus extra-hepatic metastases					
No	175	Ref		Ref	
Yes	21	1.170 (0.717-1.911)	0.530	0.966 (0.550-1.696)	0.903
AFP					
≥400 ng/mL	61	Ref		Ref	
<400 ng/mL	135	0.637 (0.462-0.879)	0.006*	0.600 (0.424-0.848)	0.004*
HbA1c[b]					
≥7 %	86	Ref		Ref	
<7 %	110	1.007 (0.742-1.367)	0.965	0.919 (0.632-1.337)	0.660
**On-sorafenib HbA1c[c]**					
≥7 %	93	Ref		Ref	
<7 %	103	0.918 (0.677-1.244)	0.581	0.870 (0.597-1.268)	0.469

Abbreviation: HR, hazard ratio; CI, confidence interval; Ref, reference variable; OHA, oral hypoglycemic agent; RI, regular insulin; NPH, neutral protamine hagedorn; HTN, hypertension; HBV, hepatitis B virus; HCV, hepatitis C virus; AFP, alpha-fetoprotein; HbA1c, hemoglobin A1c.

Note: [a]To confirm the correlation between each variable and PFS, all variables were entered into multivariate analysis. The use of RI/NPH (yes/no) was a covariate constant or linearly dependent to variables of metformin use (yes/no) and non-metformin OHA prescription (yes/no). Therefore, this variable was not entered into multivariate analysis when the other two were entered. [b]To eliminate the bias, data of baseline HbA1c level for each patient were determined by calculating the mean value of HbA1c level measured multiple (two or three) times at baseline. [c]Data of on-sorafenib HbA1c level derived from calculating the mean value of serum HbA1c level measured multiple times during sorafenib therapy. *A p-value below 0.05 was considered statistically significant.
